# Teledermatology before and after coronavirus^☆^^☆☆^

**DOI:** 10.1016/j.abd.2020.09.003

**Published:** 2021-01-30

**Authors:** Dimitri Luz Felipe Silva, Luiz Gameiro, Juliana Yumi Massuda, Renata Ferreira Magalhães, Andrea Fernandes Eloy da Costa França

**Affiliations:** Dermatology Discipline, Universidade Estadual de Campinas, Campinas, SP, Brazil

Dear editor,

The COVID-19 pandemic promoted a digital acceleration, resulting in the regulation of the use of telemedicine in Brazil. Faced with the explosion of transmission in Brazil since February 2020, in March the Federal Council of Medicine recognized the possibility of using telemedicine for the purpose of tele-guidance, tele-monitoring, and tele-consultation (CFM 1756/2020). The guidelines for remote consultations were regulated on a temporary basis soon thereafter (Ordinance No. 467 of the Ministry of Health), followed by Law 13,989, signed by the President of the Republic in April.^1^, ^2^

Despite the worldwide trend, teledermatology was still viewed with reservations in Brazil. The first experiences in this field date from the early 2000s and focused on teletriage and tele-education. Telemedhansen, Telederma, and Anapec were some of the programs in the dermatology field. More recently, a study carried out at the Telemedicine and Telehealth Center in the state of Santa Catarina allowed the diagnostic teletriage of skin cancer through the remote sharing of clinical and dermoscopic images of skin lesions.^3^, ^4^, ^5^, ^6^

To date, the adoption of this type of care by dermatologists has been seldom considered. In order to assess the adoption of dermatological tele-consultation in the private sector, the authors conducted a survey with members of the 5^th^ dermatological district of the Brazilian Society of Dermatology (Socidedade Brasileira de Dermatologia [SBD] – São Paulo Region), by sending an online form, distributed in June 2020, two months after the beginning of the lockdown. Of the total of 300 dermatologists who received the questionnaire by e-mail, located in 35 cities in the countryside of the state of São Paulo, 84 forms were returned (30% of the members).

A total of 60% of the respondents were between 41 and 60 years old. Of these, only 7% carried out tele-consultations before the pandemic and regulations.

Among those who were not conducting tele-consultations at the time of the study (n = 42), several reasons were cited; the answer “I do not feel it will meet my patient's expectations” was mentioned by 17% of the interviewees. Frustrating previous experiences (12%) and resistance to the use of technology on a daily basis (12%) were other justifications. Financial issues were also mentioned on a smaller scale (smaller payments from health insurance companies, degradation of the profession, lack of demand).

In turn, the performance of telemedicine “at the request of the patient” was the reason for the adoption of this technology by 33% of dermatologists, followed by this being “another source of income” (18%) and “decreased flow of patients in the office” (12%). The enthusiasts of this technology (early adopters) corresponded to 9% of the interviewees.^7^

When asked about the degree of resolvability of teledermatology on a scale of 1 (no resolution) to 5 (complete resolution), 53% of dermatologists rated it 3 and 33% rated it 4, indicating that 86% considered the technology capable of meeting their demands. Finally, 63% of respondents intended to continue using telemedicine, even after the end of the COVID-19 pandemic (Fig. 1).

**Figure 1:**
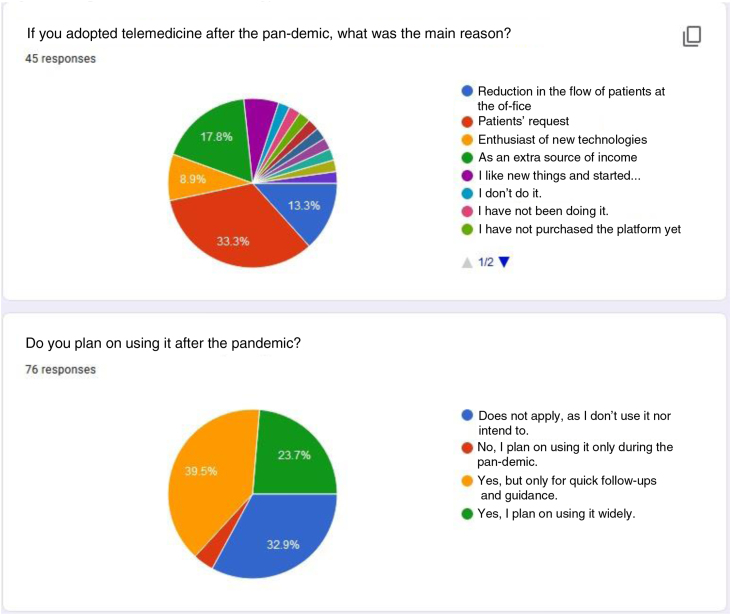
Opinion of teledermatology users.

Despite being precipitated by an exceptional situation, the scenario described in the present study reflects a tendency for a paradigm shift in the exercise of the specialty in Brazil, which may now consider teledermatology as a viable option in patient care. Considering that the majority of dermatologists who tested the new technology see it as a satisfactory tool, replicating this method to those who do not yet practice it may be one of the main obstacles in implementing this modality *a posteriori*. Financial limitations and the establishment of well-defined rules of use also appear to be important factors for its definitive implementation.

Several studies in the literature point out the effectiveness of teledermatology, both in the early detection of malignant neoplasms and in the teletriage of tegumentary complaints. Certainly, this connected healthcare model can make a difference in a continental and heterogeneous country such as Brazil.^2^, ^7^, ^8^

This survey indicated other uses of teledermatology in the private field and in the scope of offices, in addition to actions aimed at training/matrix support.

## Financial support

None declared.

## Authors’ contributions

Dimitri Luz Felipe Silva: Design and planning of the study; collection, analysis, and interpretation of data; drafting and/or critical review of the manuscript with relevant intellectual content; approval of the final version.

Luiz Gameiro: Design and planning of the study; collection, analysis, and interpretation of data; drafting and/or critical review of the manuscript with relevant intellectual content; approval of the final version.

Juliana Yumi Massuda: Design and planning of the study; collection, analysis, and interpretation of data.

Renata Ferreira Magalhães: Design and planning of the study; collection, analysis, and interpretation of data; approval of the final version.

Andrea Fernandes Eloy da Costa França: Design and planning of the study; collection, analysis, and interpretation of data; drafting and/or critical review of the manuscript with relevant intellectual content; approval of the final version.

## Conflicts of interest

None declared.
